# NOTCH3 promotes malignant progression of bladder cancer by directly regulating SPP1 and activating PI3K/AKT pathway

**DOI:** 10.1038/s41419-024-07241-0

**Published:** 2024-11-18

**Authors:** Changxue Liu, Huaixi Ge, Chengquan Shen, Ding Hu, Xinzhao Zhao, Ruize Qin, Yonghua Wang

**Affiliations:** 1 Department of Urology, The Affiliated Hospital of Qingdao University, Qingdao, Shandong China; 2 Urinary Diseases Clinical Medical Research Center of Qingdao, Qingdao, Shandong China; 3 Shandong Province Medical and Health Key Laboratory of Urology, Qingdao, Shandong China

**Keywords:** Oncogenesis, Bladder cancer

## Abstract

The biological role and precise molecular mechanisms of Notch receptor 3 (NOTCH3) in the malignant progression of bladder cancer (BLCA) remain unclear. In this study, we found that NOTCH3 was significantly upregulated and associated with poor prognosis in BLCA patients. Functional experiments demonstrated that NOTCH3 knockdown inhibited BLCA cell proliferation, migration, invasion and significantly suppressed tumor growth and metastasis in vivo as well. Mechanically, chromatin immunoprecipitation and dual-luciferase reporter assays confirmed that NOTCH3 could promote the transcription of secreted phosphoprotein 1 (SPP1), a potential downstream target gene of NOTCH3, by binding to the CSL elements in the SPP1 promoter. Moreover, we also found that targeting NOTCH3 inhibited BLCA growth and metastasis by suppressing the SPP1-PI3K/AKT axis. Our study highlights the critical role of NOTCH3-SPP1-PI3K/AKT axis in the malignant progression of BLCA, suggesting that NOTCH3 may be a potential therapeutic target for BLCA.

## Introduction

Bladder cancer (BLCA) is the second most common malignant tumor of the urinary system worldwide, which predominantly affecting males. It has high incidence and mortality rates, with an estimated 500,000 new cases and 200,000 deaths globally [1–4]. BLCA is broadly classified into non-muscle invasive bladder cancer (NMIBC) and muscle-invasive bladder cancer (MIBC) [5]. Although the prognosis for NMIBC patients has significantly improved, its propensity for multicentric occurrence and recurrence leads to recurrence in 60% of NMIBC patients and progression to MIBC in over 20% of cases [6–8]. Therefore, identifying new oncogenes that drive the aggressive progression of BLCA and elucidating the underlying molecular mechanisms is crucial.

Proteins encoded by Notch genes act as transmembrane receptors involved in various biological processes, including cell fate determination, embryonic development, organ formation, and tissue repair [9]. In mammals, there are four Notch receptors and five Notch ligands [10]. Typically, ligand activation of Notch receptors results in three cleavage events, leading to the release of the Notch intracellular domain (NICD). Subsequently, the NICD translocates to the nucleus, where it binds to CSL elements in the promoters of certain genes, initiating the transcription of downstream target genes [11]. Notably, increasing evidence indicates that dysregulation of the Notch signaling pathway intricately controls the development and progression of various solid tumors, including breast cancer [12], hepatocellular carcinoma [13], colorectal cancer [14], and gastric cancer [15]. However, the expression and function of Notch receptors in the malignant progression of BLCA remain unclear.

In this study, we confirmed that NOTCH3 is highly expressed and is associated with poor prognosis in BLCA patients. Our research indicates that NOTCH3 promotes the transcription of secreted phosphoprotein 1 (SPP1) by directly binding to the CSL elements of its promoter. Moreover, aberrant activation of the PI3K/AKT pathway by SPP1 leads to the malignant progression of BLCA. Our findings emphasize the critical role of NOTCH3 in the progression of BLCA and propose NOTCH3 as a potential therapeutic target for BLCA.

## Materials and methods

### Clinical samples

From the TCGA database (https://tcga-data.nci.nih.gov/tcga/) and the GEO database (https://www.ncbi.nlm.nih.gov/geo/query/acc.cgi?acc=GSE37815,https://www.ncbi.nlm.nih.gov/geo/query/acc.cgi?acc=GSE13507), we downloaded the mRNA expression profiles of BLCA patients. A total of 40 fresh paired samples were collected from patients undergoing radical cystectomy at the Affiliated Hospital of Qingdao University (Qingdao, Shandong, China). All experiments were conducted in accordance with the Declaration of Helsinki. Participants were informed about the study and provided consent before sample collection. This study was approved by the Ethics Committee of the Affiliated Hospital of Qingdao University.

### Cell culture

Human BC cell lines T24 and 5637, and the normal human urothelial cell line SV-HUC-1, were purchased from the Cell Bank of the Chinese Academy of Sciences (Shanghai, China). All cells were cultured in DMEM (Gibco, USA) supplemented with 10% FBS (Hyclone, USA) and 1% penicillin/streptomycin (Invitrogen, USA) in a humidified atmosphere with 5% CO2 at 37 °C. Mycoplasma contamination was checked using the MycoAlert Mycoplasma Detection Kit.

### Real-time quantitative PCR analysis (RT-qPCR)

Total RNA was extracted from cultured cells using TRIzol reagent (Invitrogen, USA) according to the manufacturer’s instructions. RNA concentration was measured using the NanoDrop ND-2000 (USA). Subsequently, total RNA was reverse transcribed into cDNA using the PrimeScript™ RT Reagent Kit (Perfect Real Time) (Takara, Japan). RT-qPCR was performed on a Roche LightCycler 480II Real-Time PCR Detection System (Roche, Basel, Switzerland), and relative mRNA expression levels were calculated using the 2 − ΔΔCt method normalized to GAPDH levels. Primers were synthesized by BGI (Beijing, China), with details provided in the Supplementary Table S1.

### Western blot

Total protein was obtained from BLCA tissues or BLCA cells and subjected to SDS-PAGE as previously described [16]. Primary antibodies were incubated overnight at 4 °C, followed by incubation with HRP-conjugated secondary antibodies for 1 h at room temperature. Immunoreactivity was detected using chemiluminescence. The results were analyzed by ImageJ software to obtain the optical density ratio of the target proteins relative to β-Actin. The antibodies utilized in this study, along with their respective vendors, product numbers, and dilutions, are listed in Supplementary Table S2.

### Immunohistochemistry

Immunohistochemistry (IHC) was performed to evaluate the expression of NOTCH3 and SPP1 proteins in BLCA patient tissues, as described in previous studies [17]. Images were captured and analyzed using an Olympus fluorescence microscope and AxioVision image analysis software. Immunoreactive score was obtained as a product of multiplication between positive cells proportion score (0–4: 0: 0%, 1: 1–25%, 2: 26–50%, 3: 51–75%, 4: 76–100%) and staining intensity score (0–3: 0: negative, 1: weak, 2: moderate and 3: strong).

### RNA-seq

T24 cells with validated stable knockdown of NOTCH3 and their control group were cultured in 6-well plates for 24 h, and 5 × 10^6^ T24 cells were collected (*n* = 3 per group). Cells were then lysed using TRIzol reagent (Invitrogen, USA) for RNA sequencing. RNA sequencing and bioinformatics data analysis were performed by GeneFund (Shanghai, China). The specific steps are as follows: First, mRNA was extracted using the KAPA Stranded mRNA-Seq Kit (Roche, KK84420). PolyA-tailed RNA fragments were captured using oligo-dT beads, and the captured fragments were then fragmented by heating. Subsequently, the KAPA Stranded mRNA-Seq Kit was used to reverse transcribe the mRNA into cDNA, followed by the preparation of high-throughput sequencing libraries. After passing library quality control, sequencing was performed on the Illumina NovaSeq 6000 platform (Illumina, USA). The raw data obtained from RNA-seq sequencing were processed to remove adapters and contaminants, resulting in clean data. The HISAT2 program was used to align the clean data to the reference genome, and transcript expression levels were calculated. Differential gene expression analysis was performed using the edgeR package.

### Lentiviral, small interfering RNA, and plasmid transfection

For transient transfection, NOTCH3 small interfering RNAs (si-NOTCH3#1, si-NOTCH3#2, si-NOTCH3#3) and SPP1 small interfering RNAs (si-SPP1#1, si-SPP1#2) were synthesized by GenePharma (Suzhou, China). NOTCH3 overexpression plasmids were purchased from GeneChem (Shanghai, China). Transfection of siRNAs or plasmids into cells was performed using Lipofectamine 3000 (Invitrogen, USA) according to the manufacturer’s instructions. To generate stable cell lines, lentiviral particles for NOTCH3 knockdown and SPP1 overexpression were obtained from GeneChem. T24 and 5637 cells were infected with lentiviral solutions to establish stable NOTCH3 knockdown, SPP1 overexpression, and matched control cell lines. The corresponding sequences are shown in Supplementary Table S3.

### Cell counting kit-8 (CCK-8) assay

BLCA cells were cultured in 96-well plates (2000 cells/well) for designated times. According to the manufacturer’s instructions, 10 μL of CCK-8 solution was added to each well and incubated at 37 °C for 3 h. Absorbance at 450 nm was measured.

### Colony formation assay

BLCA cells (500 cells/well) were plated in 6-well plates with medium containing 10% FBS and cultured for approximately 2 weeks. Colonies were fixed with 4% paraformaldehyde for 30 min and stained with 0.1% crystal violet for 30 min. Images were captured using a high-definition digital camera.

### Wound healing assay

Cells were seeded in 6-well plates, and a sterile plastic pipette tip was used to scratch the cell layer. Cells were then cultured in FBS-deficient medium, and images were taken at 0 h, 24 h, and 48 h using an electron microscope. The migration ability of the cells was evaluated by measuring changes in the wounded area.

### Transwell assay

Transwell chambers (Corning, 8 μm) with or without Matrigel were used to assess the invasion or migration abilities of BLCA cells. Briefly, 5 × 10^4^ cells were seeded into the upper chamber with 200 μL of FBS-deficient medium, while 500 μL of medium containing 10% FBS was added to the lower chamber. After 24 h of incubation at 37 °C, chambers were washed with PBS and fixed with 4% paraformaldehyde for about 30 min. Cells on the upper side of the membrane were scraped off with a cotton swab, and the membrane was stained with crystal violet for about 30 min at room temperature. The membrane was washed in PBS, dried, and images were taken using an electron microscope.

### In vivo tumor growth and metastasis assays

For the subcutaneous xenograft model, 5 × 10^6^ stably transfected cells (*n* = 6 per group) were injected subcutaneously into the abdominal region of 4–5-week-old female BALB/c nude mice. Tumor size and body weight of the mice were recorded weekly. Tumor volume was calculated as: (length × width^2^)/2. Mice were euthanized 4 weeks after cell injection, and xenograft tumors were excised, weighed, and photographed. For the lung metastasis model, 1 × 10^6^ luciferase-labeled stably transfected cells were injected into the tail vein of 4–5-week-old female BALB/c nude mice. Bioluminescence imaging was performed 2 months later using an in vivo fluorescence imaging system. Mice were euthanized, lung samples were collected, fixed with 4% paraformaldehyde, and H&E staining was performed to monitor metastatic nodules. All animal experiments were approved by the Animal Care Committee of the Affiliated Hospital of Qingdao University.

### Luciferase reporter assay

The SPP1 wild-type reporter plasmid (SPP1-WT) was constructed by inserting the SPP1 promoter fragment containing the NOTCH3 binding site into the GV238 vector (GeneChem). A mutated SPP1 plasmid (SPP1-MUT) was created by further mutating the binding site. BLCA cells were co-transfected with the reporter plasmid and either NOTCH3 overexpression plasmid or NOTCH3 small interfering RNAs. Cells were collected and lysed 48 h post-transfection. Firefly and Renilla luciferase activities was measured using a dual-luciferase assay kit (RG027, Beyotime, Shanghai, China) according to the manufacturer’s instructions. Finally, the ratio of luminescence from firefly luciferase to Renilla luciferase was calculated.

### Chromatin immunoprecipitation

Cells were fixed with 1% formaldehyde. After cell lysis, chromatin was fragmented by sonication. Antibodies and fragmented chromatin were incubated overnight at 4 °C. Protein A and G-conjugated magnetic beads (HY-K0202, MedChemExpress) were then added and incubated for 1–2 h. Beads were washed, and cross-linking was reversed, followed by DNA purification. Finally, the purified DNA was analyzed by qPCR. The primers used for qPCR are listed in Supplementary Table S3.

### Statistical analyses

Data were statistically analyzed using GraphPad prism 9.0. Data are presented as mean ± standard error of the mean (SEM) from three independent experiments. Comparisons were made using Student’s *t*-test or one-way analysis of variance (ANOVA). The correlation between NOTCH3 and SPP1 expression was analyzed using Spearman’s correlation test. Survival differences between groups were compared using Kaplan-Meier (KM) survival analysis. Statistical significance was set at *P* < 0.05. *P < 0.05, **P < 0.01, ***P < 0.001. NS not significant.

## Result

### NOTCH3 is upregulated in BLCA and associated with poor prognosis

**Fig. 1:**
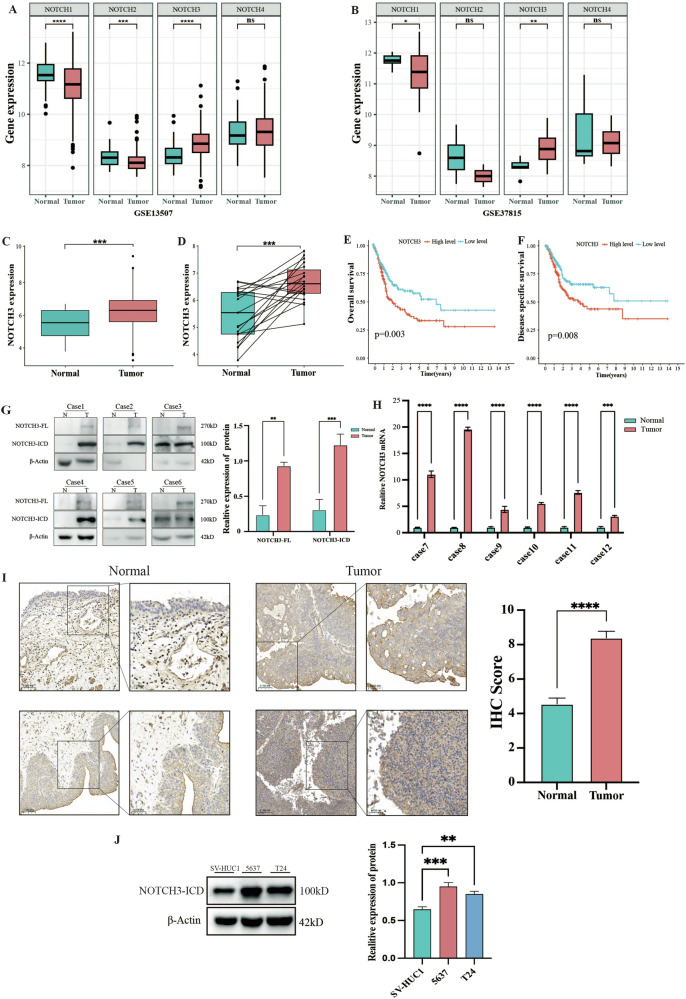
NOTCH3 is upregulated in BLCA and associated with poor prognosis. **A**, **B** The expression levels of four Notch pathway ligands in tumor and normal tissues in the GSE13507 and GSE37815 datasets; **C, D** The expression of NOTCH3 in tumor and normal tissues in the TCGA-BLCA cohort; **E**, **F** Comparison of overall survival and disease-specific survival between high and low NOTCH3 expression in the TCGA-BLCA cohort; **G**, **H** Protein and mRNA expression levels of NOTCH3 in BLCA tissues and adjacent normal tissues; **I** Representative images and quantification of NOTCH3 protein immunohistochemical staining in BLCA tissues (*n* = 40) and normal tissues (*n* = 40), scale bar: 50 μm; **J** Western blot analysis of NOTCH3 protein levels in SV-HUC1, T24, and 5637 cells. Data are presented as the mean ± SEM of three independent experiments, *p < 0.05, **p < 0.01, ***p < 0.001, ****p < 0.0001.

To elucidate the expression of Notch family receptors (NOTCH1-NOTCH4) in BLCA, we analyzed BLCA cohorts from the GEO (GSE13507 and GSE37815). However, the results indicated that NOTCH1 and NOTCH2 were downregulated, while NOTCH4 showed no statistically significant difference, and only NOTCH3 was upregulated in BLCA (Fig. 1A, B). Subsequently, we analyzed the expression of NOTCH3 in BLCA using the TCGA database. The results indicated that only NOTCH3 is upregulated in BLCA (Fig. 1C, D) and is closely associated with poor prognosis in BLCA patients (Fig. 1E, F). Therefore, NOTCH3 was selected as the target-of-interest in our following validation experiments. This finding was further validated by RT-qPCR and western blot assays in fresh BLCA tissues and adjacent non-cancerous tissues. The results showed that NOTCH3 was highly expressed and NICD expression was also elevated in BLCA (Fig. 1G, H). Additionally, similar results were obtained through immunohistochemistry in our BLCA cohort (40 samples) (Fig. 1I). Furthermore, compared to immortalized human urothelial cells (SV-HUC1), the protein level of NOTCH3-ICD was significantly elevated in BLCA cell lines (T24, 5637) (Fig. 1J). In summary, these results suggest that NOTCH3 is highly expressed in BLCA and is associated with poor prognosis.

### NOTCH3 promotes tumor growth and metastasis in BLCA

**Fig. 2:**
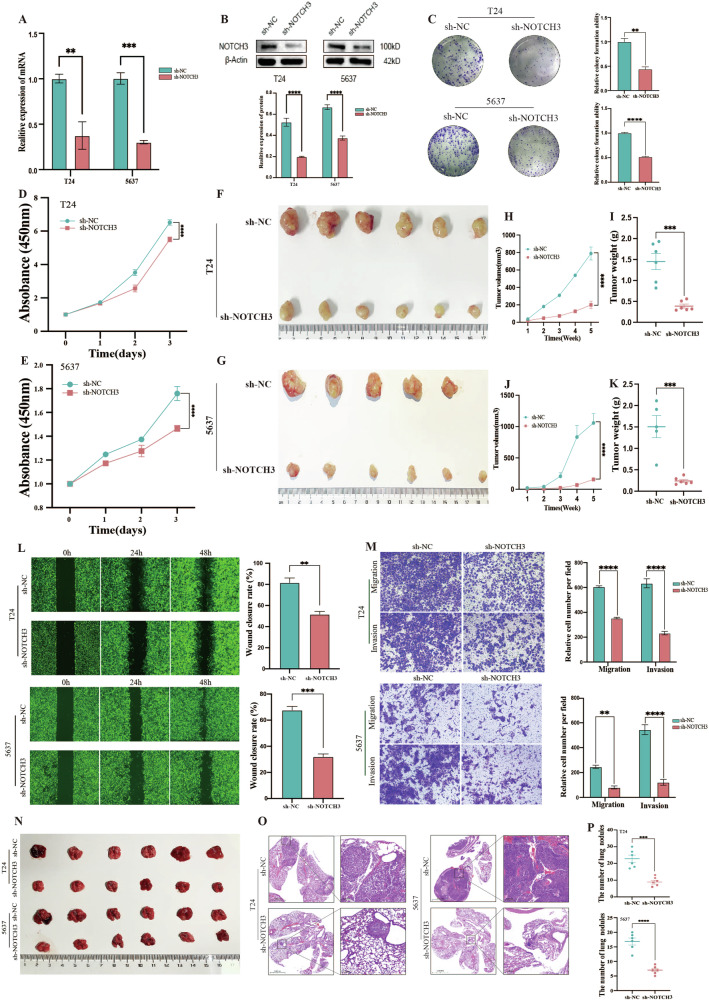
NOTCH3 promotes tumor growth and metastasis in BLCA. **A**, **B** The knockdown efficiency of NOTCH3 in T24 and 5637 cells was determined by qRT-PCR and Western blot analysis. **C** Clonogenic assay evaluating the proliferation ability of T24 and 5637 cells after NOTCH3 knockdown. **D**, **E** Cell proliferation assessed by CCK8 assay. T24 and 5637 cells transfected with sh-NC and sh-NOTCH3 were subcutaneously injected into the groin of BALB/c-nude mice (*n* = 6), and tumor size (**F**, **G**), volume (**H**, **J**), and weight (**I**, **K**) were measured 5 weeks later. **L** Wound healing assay assessing the migration ability of T24 and 5637 cells after NOTCH3 knockdown. **M** Transwell assay evaluating the invasion and migration abilities of T24 and 5637 cells after NOTCH3 knockdown. T24 and 5637 cells transfected with sh-NC and sh-NOTCH3 were injected into the tail vein of nude mice to establish a lung metastasis model, observed 5 weeks later. **N** Lung tissue was observed, and (**O**) lung nodules were quantified using HE staining, followed by statistical analysis (**P**). Data are presented as the mean ± SEM of three independent experiments, *p < 0.05, **p < 0.01, ***p < 0.001, ****p < 0.0001.

To further investigate the biological function of NOTCH3 in BLCA, we screened three small interfering RNAs (siRNAs) and selected the one with the highest knockdown efficiency to construct a stable NOTCH3 knockdown cell line (Supplementary Fig. S1). The knockdown efficiency of NOTCH3 was confirmed at both the mRNA and protein levels (Fig. 2A, B). CCK-8 assays demonstrated that knockdown of NOTCH3 significantly inhibited tumor cell proliferation (Fig. 2D, E), and similar results were obtained from colony formation assays (Fig. 2C). Subsequently, a subcutaneous xenograft model was established in BALB/c-nude mice, and the results showed that knockdown of NOTCH3 significantly reduced tumor volume and weight, indicating a crucial role of NOTCH3 in tumor growth (Fig. 2F–K). Moreover, Transwell and wound healing assays confirmed that the migration and invasion abilities of NOTCH3 knockdown BLCA cells were considerably diminished (Fig. 2L, M). The lung metastasis model of BLCA further confirmed that NOTCH3 knockdown inhibited tumor metastasis in mice (Fig. 2N–P). These findings suggest that NOTCH3 plays a significant role in the growth and metastasis of BLCA.

### NOTCH3 activates SPP1 transcription by directly binding to CSL binding elements in BLCA

**Fig. 3:**
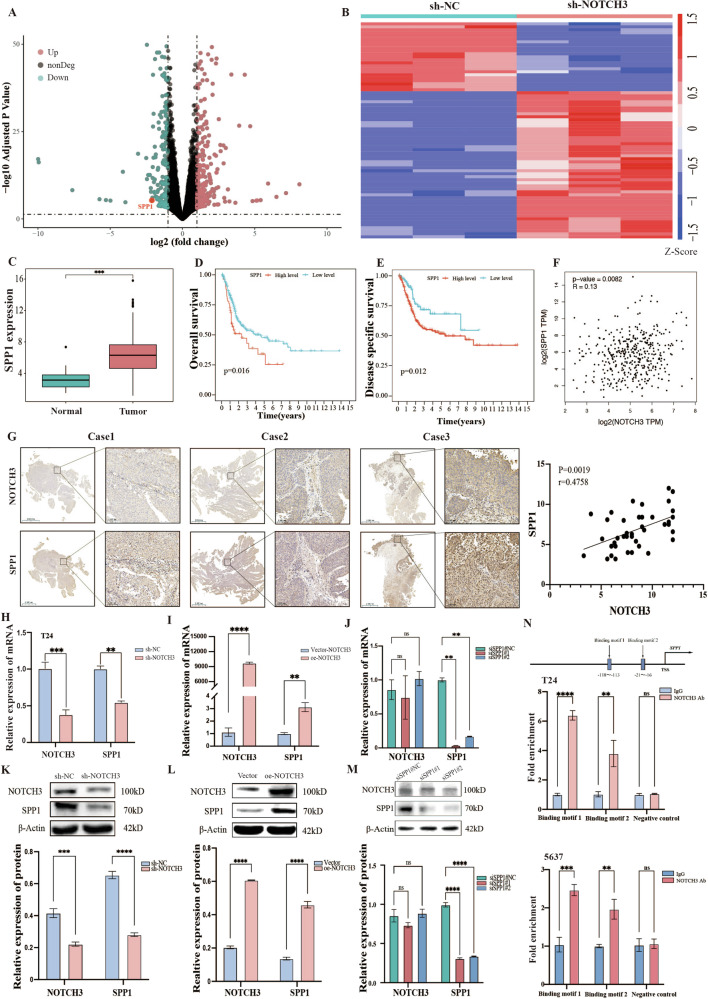
NOTCH3 activates SPP1 transcription by directly binding to CSL binding elements in BLCA. **A** Volcanic and (**B**) heatmap of differentially expressed genes by RNA sequencing in T24 cells of sh-NOTCH3 and sh-NC (*n* = 3). **C** Expression of SPP1 in tumor and normal tissues from the TCGA-BLCA cohort. **D**, **E** Comparison of overall survival and disease-specific survival between high and low expression of SPP1 in the TCGA-BLCA cohort. **F** Positive correlation between NOTCH3 and SPP1 expression as shown in the GEPIA database. **G** Representative immunohistochemical staining images of NOTCH3 and SPP1 proteins in BLCA tissues (*n* = 40) and adjacent normal tissues (*n* = 40). Spearman correlation analysis of NOTCH3 and SPP1 protein expression in BLCA specimens. **H**–**M** Changes in mRNA and protein levels of SPP1 after knockdown or overexpression of NOTCH3 detected by qRT-PCR and Western blot, and changes in mRNA and protein levels of NOTCH3 after knockdown of SPP1. **N** Enrichment of NOTCH3 at two binding sites in the SPP1 promoter detected by ChIP-qPCR. Data are presented as the mean ± SEM of three independent experiments, *p < 0.05, **p < 0.01, ***p < 0.001, ****p < 0.0001.

To elucidate the molecular mechanisms of NOTCH3 promoting the malignant progression of BLCA, RNA sequencing was performed on T24 cells with NOTCH3 knockdown, revealing 422 differentially expressed genes positively correlated with NOTCH3 (*P* < 0.05, |fold change| >1) (Fig. 3A, B). To identify the potential target genes through which NOTCH3 promotes malignant progression in BLCA, we analyzed the expression of the top 20 downregulated differential genes (Supplementary Table S4) using TCGA databases, as well as their association with the prognosis of BLCA patients. Among the 20 genes, only SPP1 was highly expressed in BLCA patients and closely associated with poor prognosis, thus identifying SPP1 as a potential interactor of NOTCH3 (Fig. 3B–D). Subsequently, we utilized the GEPIA online database and found a strong positive correlation between NOTCH3 and SPP1 (Fig. 3F). To further validate the correlation between NOTCH3 and SPP1 in BLCA, we performed immunohistochemistry and found a positive correlation between NOTCH3 and SPP1 expression in BLCA tissues (Fig. 3G). We then evaluated SPP1 mRNA and protein levels in BLCA cell lines with NOTCH3 knockdown or overexpression. The results showed that SPP1 expression paralleled NOTCH3 expression, with high levels of SPP1 observed in NOTCH3 overexpressing cells and downregulated levels of SPP1 observed in NOTCH3 knockdown cells (Fig. 3H, I, K, L) (Supplementary Fig. S2A, B, D, E). Furthermore, γ-secretase inhibitor (DAPT) inhibited NOTCH3 cleavage, reducing NICD and downregulating SPP1 expression (Supplementary Fig. S2C). However, silencing SPP1 with siRNA failed to alter the mRNA and protein levels of NOTCH3 (Fig. 3J, M) (Supplementary Fig. S2F, G). In summary, these results indicate a correlation between NOTCH3 and SPP1 in BLCA. Previous studies have shown that the NOTCH transcription factor family can bind to CSL promoter elements to regulate the expression of downstream target genes [11]. Thus, we investigated whether NOTCH3 could directly regulate SPP1 expression by binding to the CSL elements in its promoter. Using the JASPAR database, we predicted the binding sites of NOTCH3 in the SPP1 promoter (Region1: −118 bp to −113 bp, Region2: −21 bp to −16 bp) and designed two pairs of primers containing CSL binding elements for CHIP assays. In addition, we designed a third pair of primers as a negative control specifically for regions without CSL binding elements. The CHIP assays revealed that NOTCH3 binds to the SPP1 promoter in T24 and 5637 cells, with more significant binding at Region1; however, this binding was absent in the negative control. (Fig. 3N). To further validate the regulatory effect of NOTCH3 on SPP1, we constructed a luciferase reporter gene containing WT or mutated CSL binding elements from the SPP1 promoter. Dual-luciferase assays showed that SPP1 promoter activity was significantly inhibited in NOTCH3 knockdown cells. Conversely, NOTCH3 overexpression increased luciferase activity in cells with WT SPP1, whereas but not of those with mutated SPP1(Supplementary Fig. S2H). These results indicate that NOTCH3 promotes the transcription of SPP1 by binding to the CSL elements in the promoter.

### SPP1 activates the PI3K-AKT pathway to promote BLCA cell proliferation, migration, and invasion

**Fig. 4:**
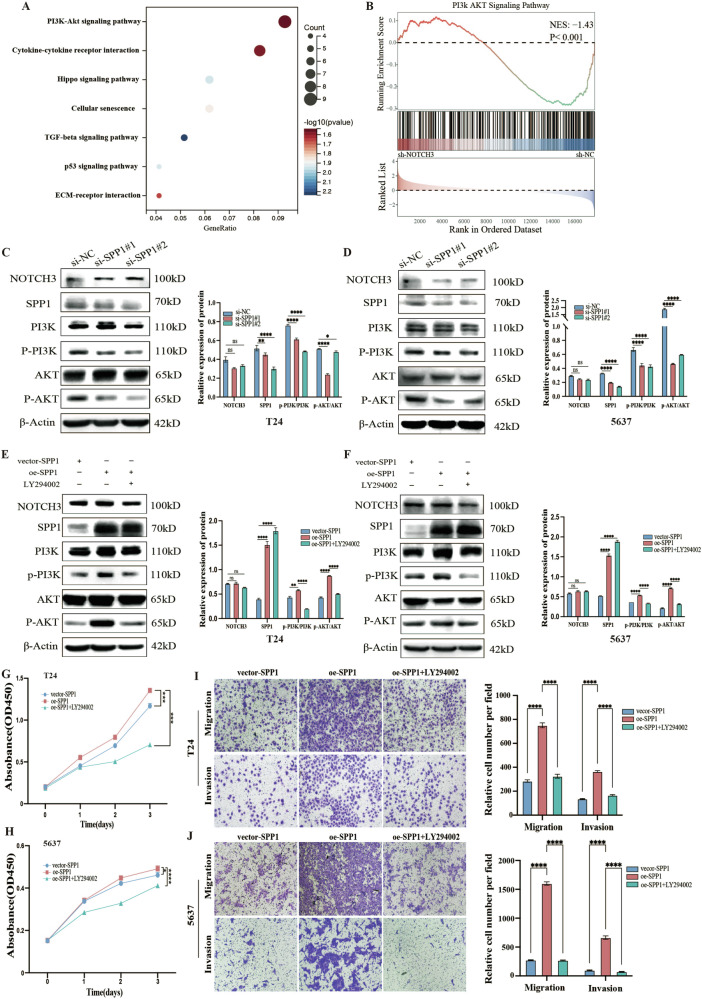
SPP1 activates the PI3K-AKT pathway to promote BLCA cell proliferation, migration, and invasion. **A** KEGG enrichment analysis revealed the molecular pathway map of differentially expressed genes, after RNA sequencing of T24 cells. **B** GSEA analysis showing significant enrichment of the PI3K/AKT pathway in RNA sequencing data. **C**, **D** Western blot analysis of changes in PI3K/AKT pathway-related proteins and their phosphorylation levels after knocking down SPP1 in T24 and 5637 cells. **E**, **F** Further validation of PI3K/AKT signaling pathway activity by treating T24 and 5637 cells overexpressing SPP1 with the PI3K-specific inhibitor LY294002, as assessed by Western blot. **G**, **H** Assessment of cell proliferation capacity using the CCK8 assay. **I**, **J** Evaluation of cell migration and invasion capabilities using the Transwell assay. Data are presented as the mean ± SEM of three independent experiments, *p < 0.05, **p < 0.01, ***p < 0.001, ****p < 0.0001.

Based on our RNA sequencing results, KEGG and GSEA pathway enrichment analyses revealed significant enrichment of the PI3K/AKT pathway (Fig. 4A, B). Previous studies have shown that the PI3K/AKT signaling pathway is a key pathway controlling cell functions in mammalian cells, and its abnormal activation can promote tumor development and progression. Therefore, we further investigated whether the PI3K/AKT pathway is a downstream activation pathway of SPP1. The Western Blot results showed that after SPP1 knockdown, the expression levels of PI3K and AKT proteins remained unchanged, but the phosphorylation levels of PI3K and AKT significantly decreased, indicating that SPP1 downregulation can inhibit the activity of the PI3K/AKT pathway (Fig. 4C, D). Next, we overexpressed SPP1 in T24 and 5637 cells and treated with the PI3K/AKT pathway inhibitor LY294002. The results showed that the phosphorylation levels of PI3K and AKT were increased after SPP1 overexpression, while that were recovered after treatment with LY294002 (Fig. 4E, F). Furthermore, CCK-8 assay results showed that SPP1 overexpression promoted tumor cell proliferation, but this effect disappeared after treatment with LY294002 (Fig. 4G, H). Transwell assays also showed that SPP1 overexpression promoted cell migration and invasion, but inhibition of the PI3K/AKT pathway negated the effects of SPP1 overexpression (Fig. 4I, J). Therefore, increased SPP1 could promotes BLCA proliferation, migration, and invasion through activation of the PI3K/AKT pathway.

### Targeting NOTCH3 could effectively suppress tumor growth and metastasis by regulated SPP1-PI3K/AKT axis

**Fig. 5:**
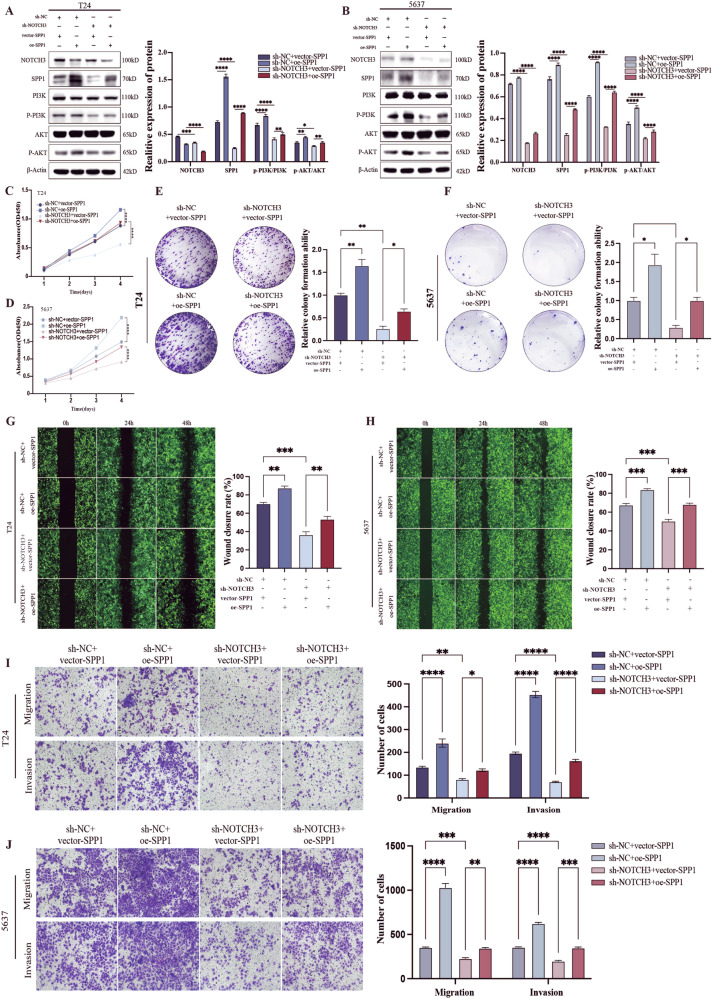
Targeting NOTCH3 can effectively inhibit the proliferation, migration and invasion of tumor cells by regulating the SPP1-PI3K/AKT axis. **A**, **B** Western blot analysis of phosphorylation levels of NOTCH3, SPP1, PI3K, and AKT in each treatment group (sh-NC+vector-SPP1, sh-NC+oe-SPP1, sh-NOTCH3+vector-SPP1, sh-NOTCH3+oe-SPP1). **C**, **D** Assessment of proliferation capacity of stable transfected T24 and 5637 cells using the CCK8 assay. **E**, **F** Clonogenic assay to evaluate proliferation capacity of T24 and 5637 cells post-treatment. **G**, **H** Representative images and statistical analysis of wound healing assay assessing migration ability of T24 and 5637 cells. **I**, **J** Representative images and statistical analysis of Transwell assay evaluating invasion and migration capabilities of T24 and 5637 cells. Data are presented as the mean ± SEM of three independent experiments, *p < 0.05, **p < 0.01, ***p < 0.001, ****p < 0.0001.

**Fig. 6:**
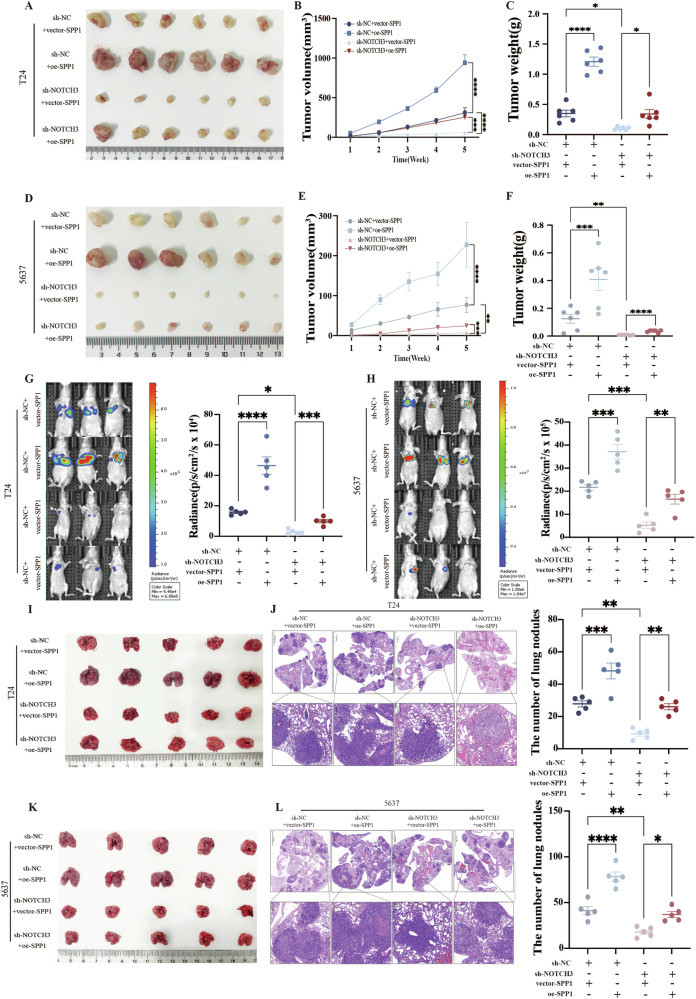
Targeting NOTCH3 could effectively suppress tumor growth and metastasis by regulated SPP1-PI3K/AKT axis. Targeted NOTCH3 T24 cells (**A**) and 5637 cells (**D**) were subcutaneously injected into the inguinal region of nude mice (*n* = 6). Tumor volume (**B**, **E**) and weight (**C**, **F**) were measured after 5 weeks. Targeted NOTCH3 T24 cells (**G**) and 5637 cells (**H**) were injected into the tail vein of mice, and bioluminescence imaging was used to statistically analyze the luminescence intensity in the lungs of the mice. The lungs were harvested, the number of lung nodules was counted (**I**, **K**), and the nodules in the lung tissue were subjected to HE staining (**J**, **L**). Data are presented as the mean ± SEM of three independent experiments, *p < 0.05, **p < 0.01, ***p < 0.001, ****p < 0.0001.

We further evaluated the effects of targeting NOTCH3 on tumor growth and metastasis in BLCA, as well as the regulation of SPP1-PI3K/AKT axis. First, we found that targeting NOTCH3 led to a significant downregulation of the SPP1-PI3K/AKT axis at the protein level, which was reversed upon SPP1 overexpression (Fig. 5A, B). These results suggest a regulatory role of NOTCH3 on the SPP1-PI3K/AKT axis. In vitro, CCK8 and colony formation assays showed that targeting NOTCH3 significantly inhibited the proliferation of BLCA cells (Fig. 5C–F). Additionally, Transwell and wound healing assays demonstrated that targeting NOTCH3 inhibited the migration and invasion of tumor cells (Fig. 5G–J). However, these inhibitory effects were reversed with SPP1 overexpression. In vivo, subcutaneous xenograft and lung metastasis models showed that targeting NOTCH3 reduced tumor volume and weight (Fig. 6A–F) and inhibited lung metastasis (Fig. 6G–L). Consistent with the in vitro results, the inhibitory effects of targeting NOTCH3 on tumor growth and metastasis were counteracted by SPP1 overexpression. Overall, these findings indicate that targeting NOTCH3 can inhibit tumor growth and metastasis, suggesting that NOTCH3 is a potential therapeutic target for BLCA.

## Discussion

The Notch pathway is an evolutionarily conserved signaling pathway composed of multiple receptors and ligands, with mammals having four types of Notch receptors (NOTCH1-NOTCH4) [10]. In various solid tumors, Notch acts as an oncogenic pathway involved in malignant progression. Activated Notch signaling promotes tumor growth and metastasis [18], leading to poor prognosis in patients with melanoma [19], non-small-cell lung cancer [20], and colorectal cancer [14]. Our study demonstrates that NOTCH3 is highly expressed and associated with poor prognosis in BLCA patients. Zhang et al. found that high NOTCH3 expression is related to poor survival in patients [21], which is consistent with our findings. Furthermore, our experiments showed that depletion of NOTCH3 inhibited tumor cell proliferation, migration, and invasion, and suppressed BLCA growth and metastasis in vivo. These results suggest that NOTCH3 plays a critical role in the malignant progression of BLCA.

Previous investigations have revealed that NOTCH family receptors can promote the transcription of downstream target genes by binding to CSL elements in the promoter regions [22–24]. However, the regulatory mechanisms of NOTCH in BLCA remain unclear. For the first time, our research demonstrated that NOTCH3 could directly bind to the CSL elements in the SPP1 promoter, thereby enhance SPP1 transcription and activate the PI3K/AKT pathway to promote the malignant progression of BLCA. SPP1, also known as Osteopontin, is a multifunctional protein expressed in various cell types [25]. Previous studies have demonstrated that SPP1 is overexpressed in multiple malignancies and involved in tumorigenesis and metastasis, including colorectal cancer [26], lung cancer [27], and breast cancer [28]. Nedjadi et al. found that SPP1 is differentially expressed in the early stages of BLCA and associated with poor prognosis of BLCA patients [29]. Our research confirmed a significant positive correlation between the expression of NOTCH3 and SPP1. More importantly, we found that NOTCH3 could directly bind to the CSL elements in the SPP1 promoter, promoting SPP1 transcription and activating the PI3K/AKT pathway to enhance the proliferation, migration, and invasion of BLCA cells. However, the specific regulatory mechanisms still require further investigation in the future.

Currently, Antibody-drug conjugates (ADCs) such as Enfortumab Vedotin targeting Nectin-4, Disitamab Vedotin targeting HER2, have shown promising effect in the treatment of solid tumors, including BLCA [30, 31]. PF-06650808, a novel anti-NOTCH3 ADCs, exhibited a promising antitumor activity in a clinical study of breast cancer (NCT02129205) [32]. In our study, we found that targeting NOTCH3 effectively inhibited the growth and metastasis of BLCA by downregulating of the SPP1-PI3K/AKT axis, suggesting the potential value of targeting NOTCH3 as a promising therapeutic strategy for BLCA.

In summary, NOTCH3 expression is upregulated in BLCA tissues and cell lines and in turn regulates the SPP1 and PI3K/AKT pathway via directly binding the CSL elements of promoter to promote the malignant progression of BLCA. Moreover, targeting NOTCH3 can effectively suppress BLCA growth and metastasis. Therefore, our study highlights that NOTCH3 may be a potential therapeutic target for BLCA treatment.

## Supplementary information

Supplementary Figure S1

Supplementary Figure S2

Supplementary Table

Original data

## Data Availability

The data described in this article are available in GEO database (GSE279910).
