# Supplementary figures and images for: NOTCH3 promotes malignant progression of bladder cancer by directly regulating SPP1 and activating PI3K/AKT pathway
Source: Cell Death Dis. 2024 Nov 18;15(11):840. doi: 10.1038/s41419-024-07241-0 (PMC11574029; doi:10.1038/s41419-024-07241-0)

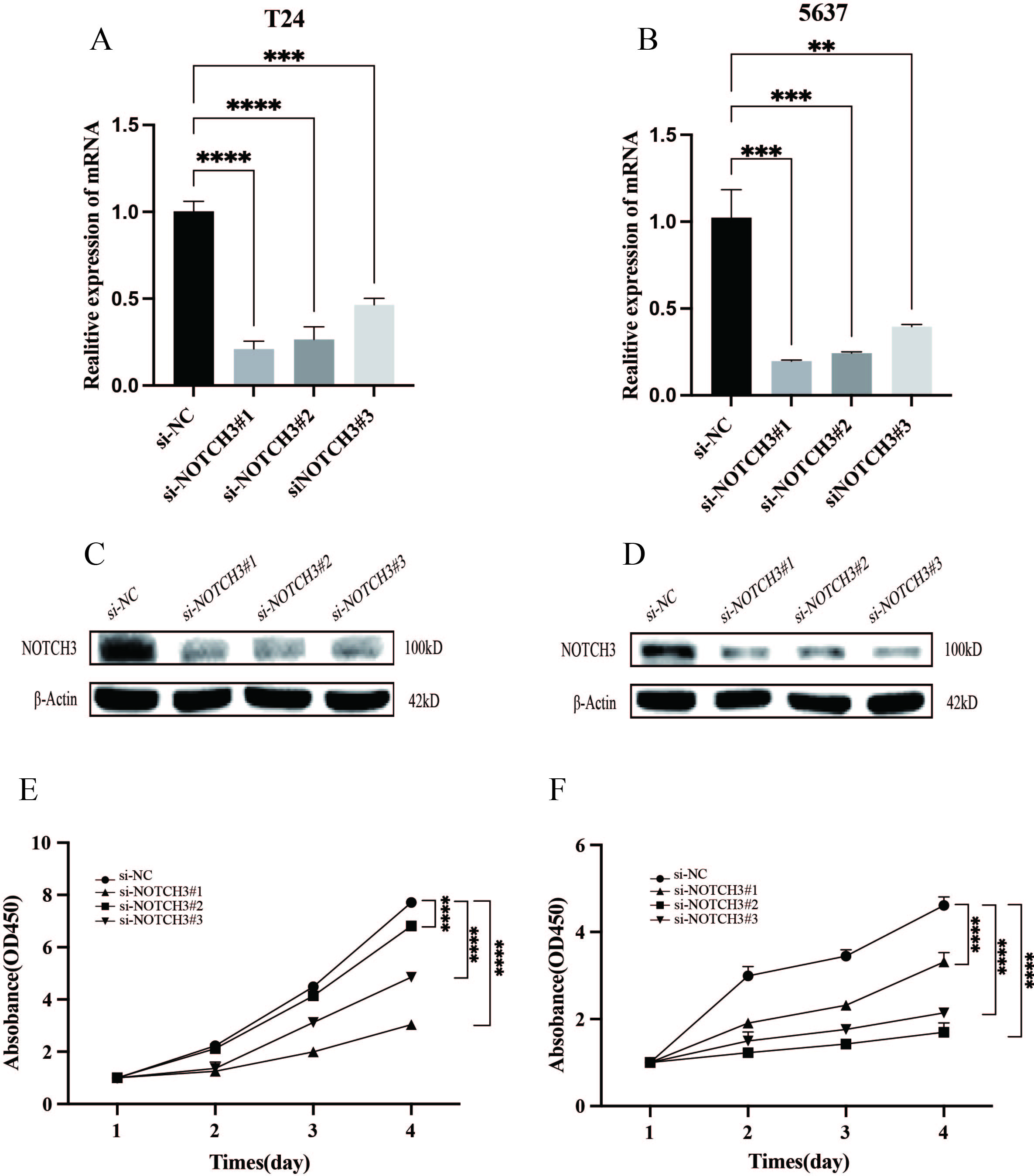

Supplement: Supplementary file 1 — Supplementary Figure S1 [file 41419_2024_7241_MOESM1_ESM.jpg]

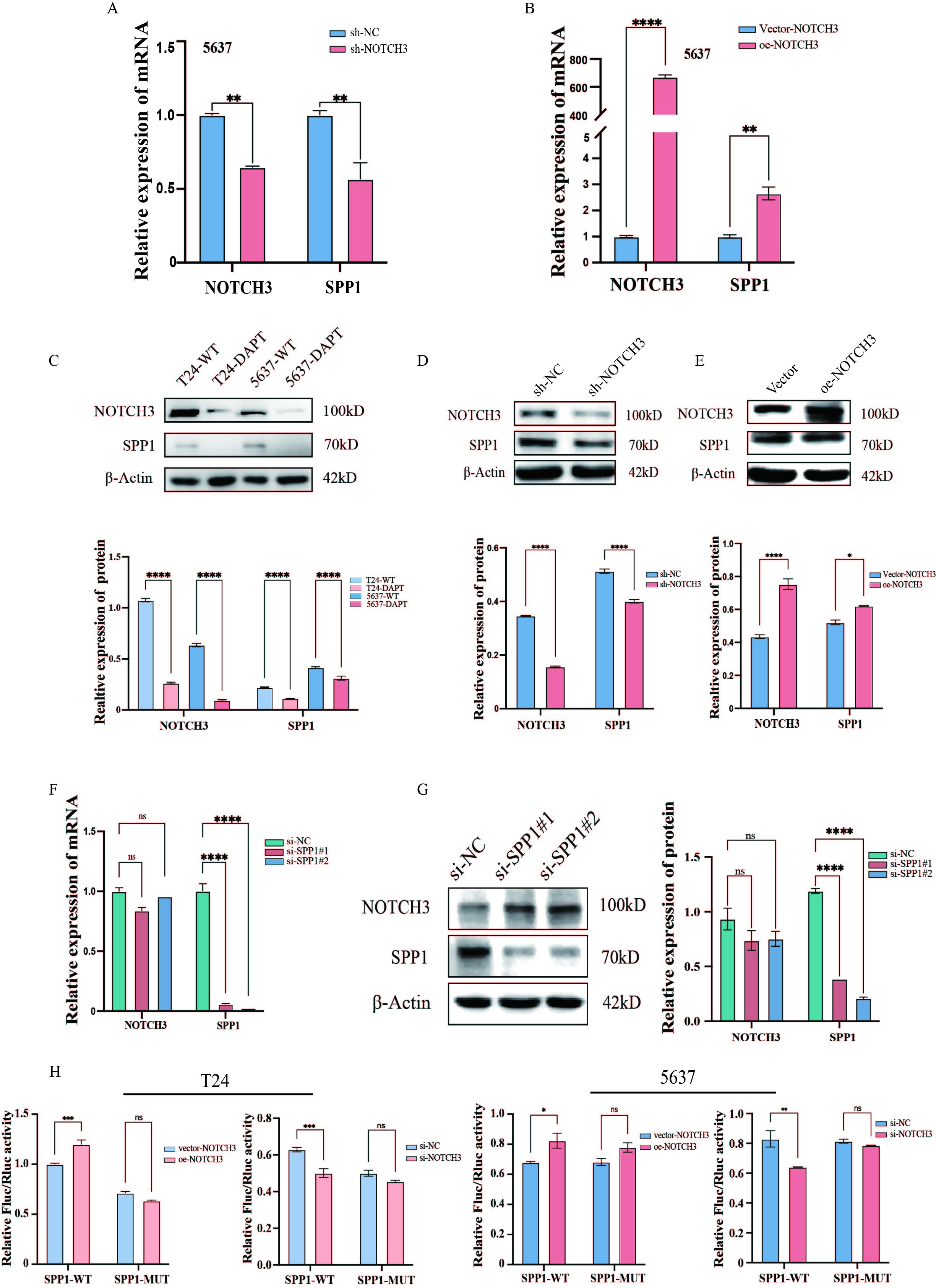

Supplement: Supplementary file 2 — Supplementary Figure S2 [file 41419_2024_7241_MOESM2_ESM.jpg]

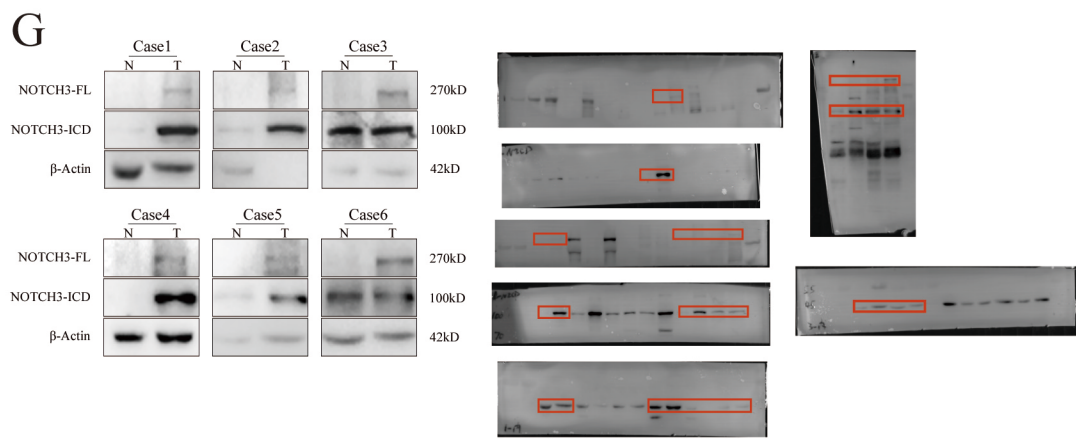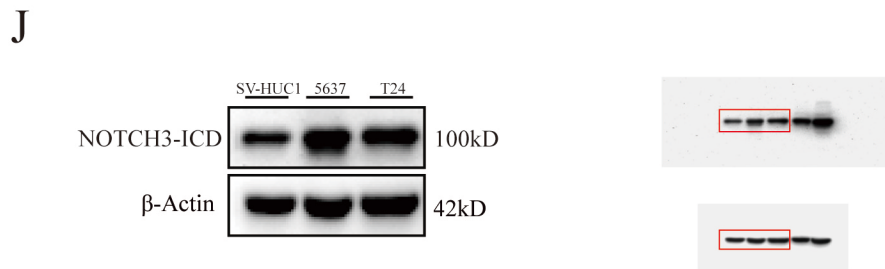

Figure1

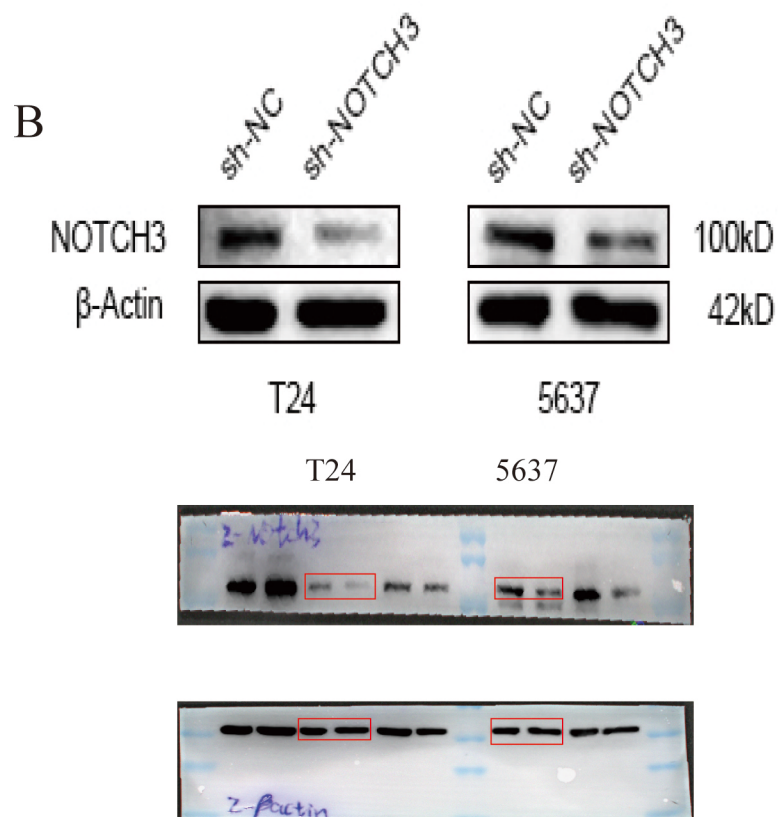

Figure2

K

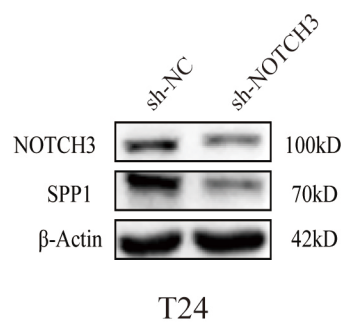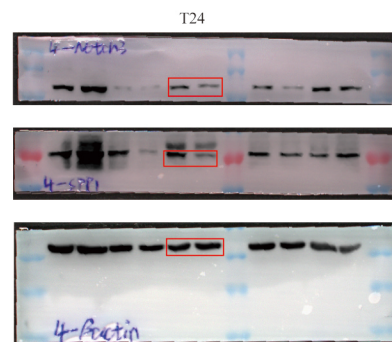

L

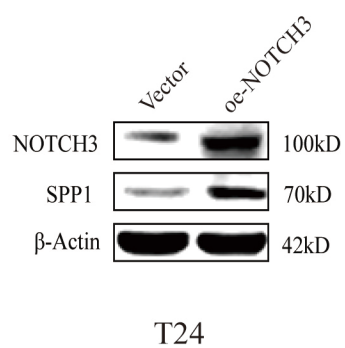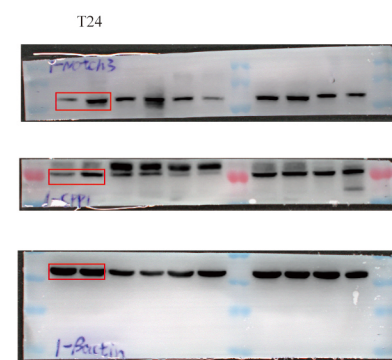

M

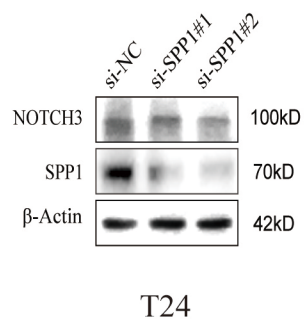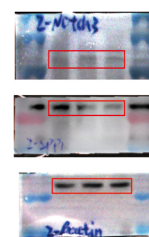

Figure3

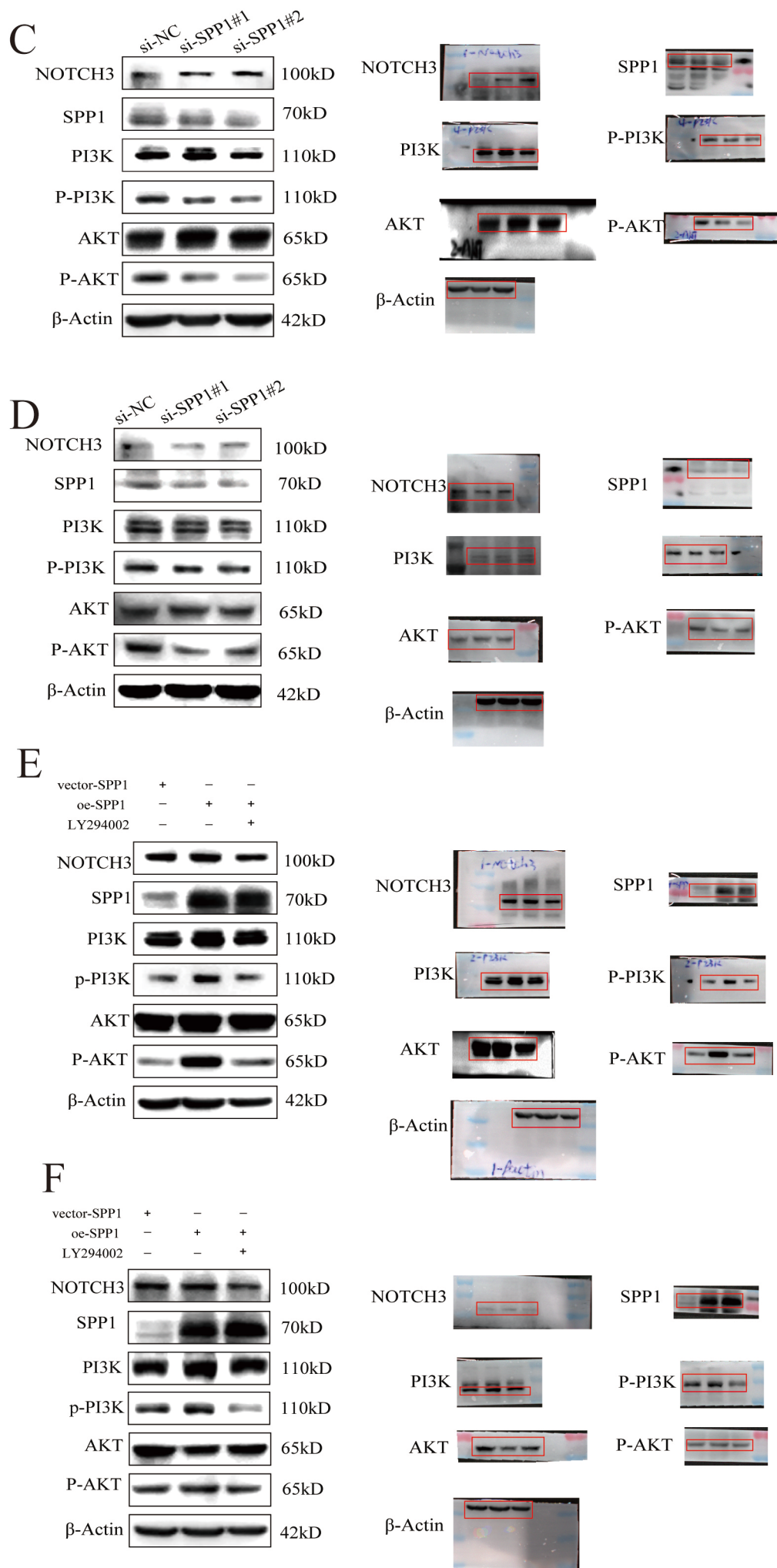

Figure4

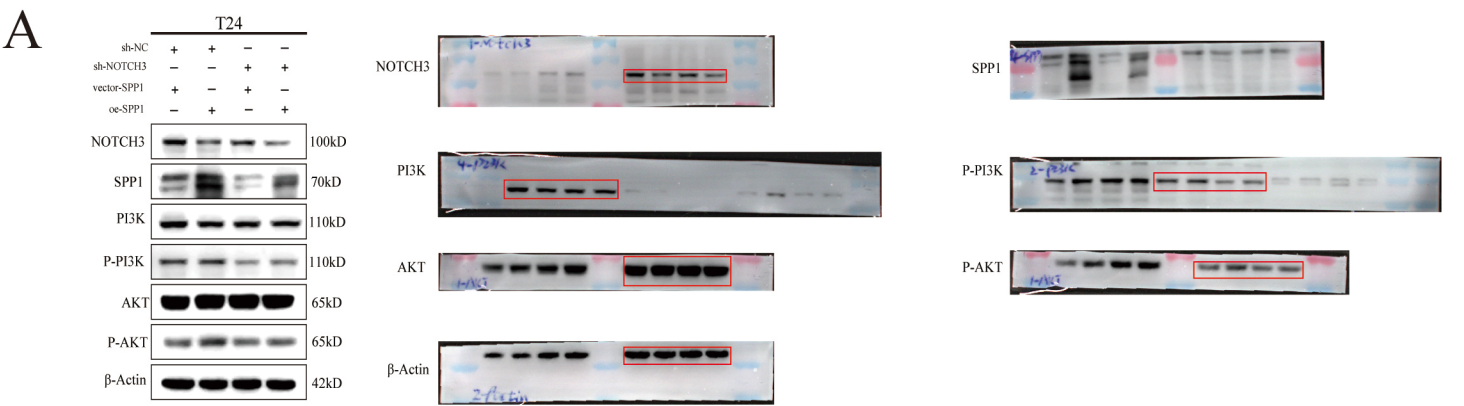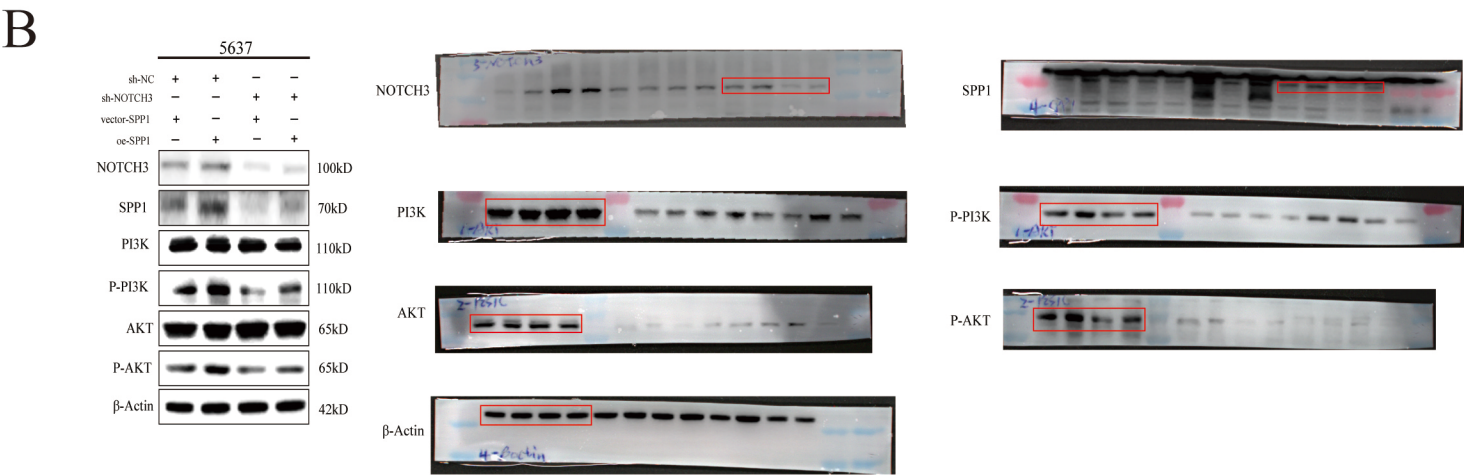

Figure5

C

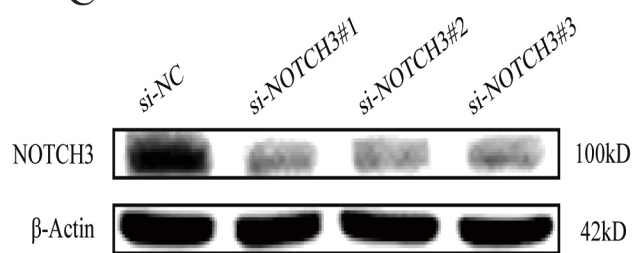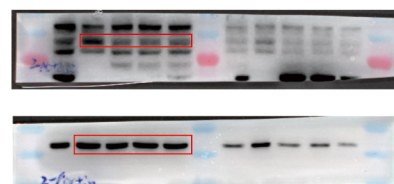

D

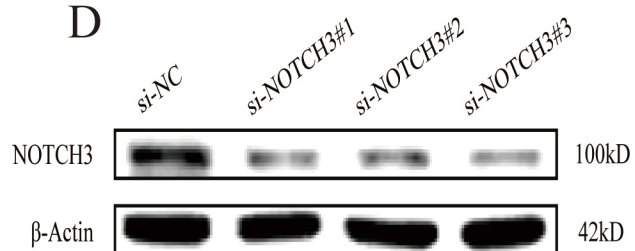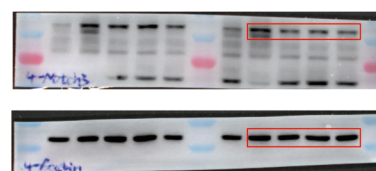

Supplementary Figure 1

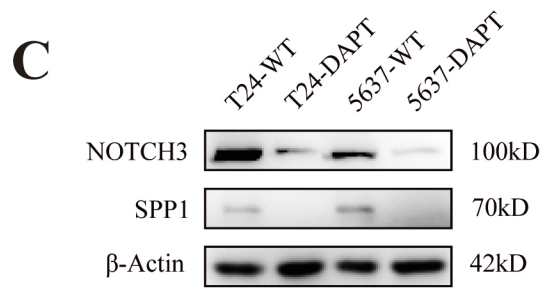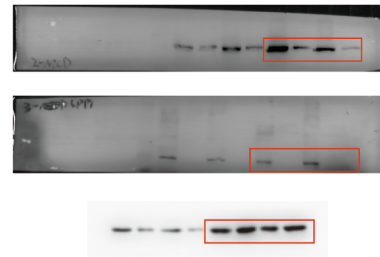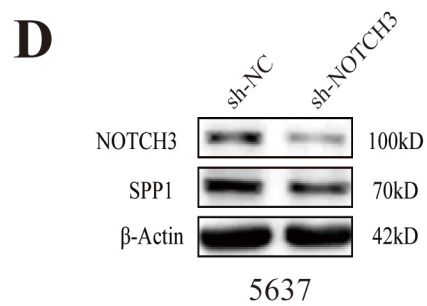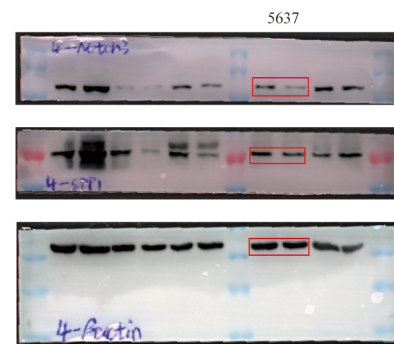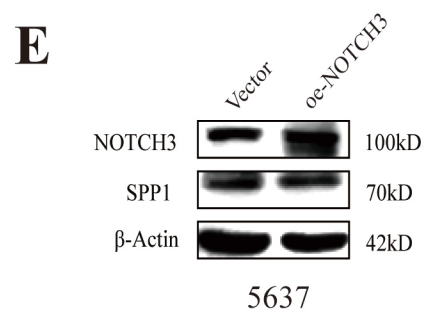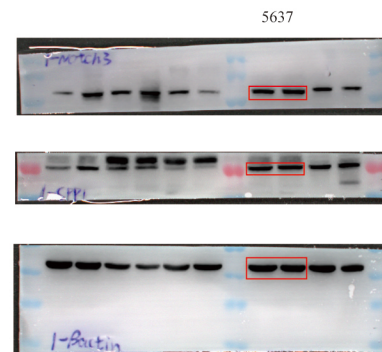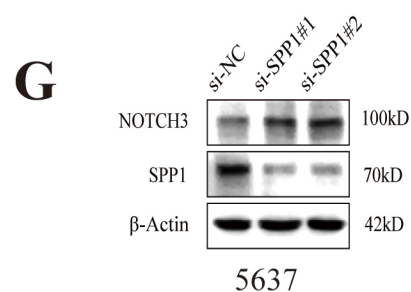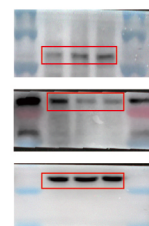

Supplementary Figure2

Supplement: Supplementary file 4 — Original data [file 41419_2024_7241_MOESM4_ESM.pdf]
