# Supplementary material for: NOTCH3 promotes malignant progression of bladder cancer by directly regulating SPP1 and activating PI3K/AKT pathway
Source: Cell Death Dis. 2024 Nov 18;15(11):840. doi: 10.1038/s41419-024-07241-0 (PMC11574029; doi:10.1038/s41419-024-07241-0)
Supplement: Supplementary file 3 — Supplementary Table [file 41419_2024_7241_MOESM3_ESM.docx]

**Table S1.** **Sequences of primers used in this study**

| Name | Sequences of Primer |
| --- | --- |
| NOTCH3-F  NOTCH3-R  SPP1-F  SPP1-R  GAPDH-F  GAPDH-R  Binding motif 1-F  Binding motif 1-R  Binding motif 2-F  Binding motif 2-R | TGGCGACCTCACTTAAGACT CACTGGCAGTTATAGGTGTTGAC CTCCATTGACTCGAACGACTC CAGGTCTGCGAAACTTCTTAGAT TCATGGGTGTGAACCATGAGAA  GGCATGGACTGTGGTCATGAG GTTGGTGGAGGATGTCTGCA CAACTGGCCTGAGACGAGTC  GTTGGTGGAGGATGTCTGCA CAACTGGCCTGAGACGAGTC |
| Negative control-F | ACCTGTTCTTTTAAAGGACATTACAA |
| Negative control-R | TGCAAATGCTCTGCGTATCT |

**Table S2. Antibodies utilized in the study**

| Antibody | Company | Cat. Number | Dilution |
| --- | --- | --- | --- |
| NOTCH3  SPP1  PI3K  p-PI3K  AKT  p-AKT  β-Actin | CST  Proteintech  Abcam  Bioss  CST  CST  CST | 5276S  22952-1-AP  ab40776  bs-5570R  4685S  4060S  3700 | 1:1000  1:1000  1:1000  1:1000  1:1000  1:2000  1:5000 |

**Table S3. The target sequences of siRNA and shRNA used in the study**

| Name | Target Sequences |
| --- | --- |
| siNOTCH3#1-F  siNOTCH3#1-R  siNOTCH3#2-F  siNOTCH3#2-R  siNOTCH3#3-F  siNOTCH3#3-R  siSPP1#1-F  siSPP1#1-R  siSPP1#2-F  siSPP1#2-R  shNOTCH3 | CCACGAGGATGCTATCTGT  ACAGATAGCATCCTCGTGG  CCATGGTCTTCCCTTACCA  TGGTAAGGGAAGACCATGG  GCGATGGAATGGGTTTCCA  TGGAAACCCATTCCATCGC  CCAGTTAAACAGGCTGATT  AATCAGCCTGTTTAACTGG  GTCTCACCATTCTGATGAA  TTCATCAGAATGGTGAGAC  CCACGAGGATGCTATCTGT |

**Table S4. The expression of the top 20 down-regulated differential genes**

| **Gene** | **sh-NOTCH3#1** | **sh-NOTCH3#2** | **sh-NOTCH3#3** | **sh-NC#1** | **sh-NC#2** | **sh-NC#3** | **logFC** | **P Value** |
| --- | --- | --- | --- | --- | --- | --- | --- | --- |
| ISY1-RAB43 | 10.12164 | 10.12164 | 10.12164 | 11.06044 | 10.69051 | 10.68882 | -9.944238 | 6.13E-17 |
| KLHL4 | 10.12164 | 10.12164 | 10.12164 | 10.41882 | 10.36747 | 10.26806 | -5.739528 | 2.32E-06 |
| LOC100505851 | 10.12164 | 10.12164 | 10.12164 | 10.40362 | 10.30551 | 10.32205 | -5.29654 | 4.07E-06 |
| GPR83 | 10.12164 | 10.12164 | 10.12164 | 10.38337 | 10.31947 | 10.31527 | -4.983426 | 5.84E-06 |
| TGFBI | 10.43313 | 10.38521 | 10.61194 | 11.85338 | 11.78466 | 11.46115 | -4.123001 | 3.29E-14 |
| SYT14 | 10.32516 | 10.39943 | 10.2981 | 10.84919 | 10.82473 | 10.77716 | -3.07149 | 2.28E-19 |
| VGLL3 | 10.40922 | 10.46159 | 10.48619 | 11.08217 | 10.95118 | 10.82013 | -2.50733 | 3.80E-18 |
| SLC2A3 | 10.74107 | 10.76867 | 10.6824 | 11.46569 | 11.59853 | 11.54161 | -2.459596 | 1.50E-50 |
| DIO2 | 10.33381 | 10.30814 | 10.34276 | 10.68679 | 10.6623 | 10.63165 | -2.455773 | 4.09465E-12 |
| NEBL | 10.32953 | 10.46719 | 10.39638 | 10.855 | 10.80494 | 10.79182 | -2.371401 | 2.77E-13 |
| THBS1 | 11.52075 | 11.54051 | 11.98846 | 13.44607 | 13.43371 | 13.15362 | -2.256314 | 2.70E-13 |
| CPA4 | 12.78513 | 12.87214 | 13.17564 | 14.98738 | 14.91635 | 14.80391 | -2.256007 | 1.4395E-100 |
| PAPPA2 | 10.35401 | 10.31325 | 10.51523 | 10.83741 | 10.78322 | 10.8033 | -2.191078 | 1.54E-09 |
| NRXN3 | 10.43883 | 10.24603 | 10.45125 | 10.82993 | 10.82352 | 10.72774 | -2.176622 | 1.69E-08 |
| SPP1 | 10.12164 | 10.29191 | 10.31026 | 10.53594 | 10.54433 | 10.57422 | -2.114219 | 4.94E-06 |
| IGFBP5 | 10.2687 | 10.28615 | 10.27807 | 10.46672 | 10.48379 | 10.60654 | -2.078698 | 8.33E-07 |
| EDIL3 | 10.47356 | 10.42898 | 10.44448 | 10.83741 | 10.93577 | 10.72451 | -2.061082 | 2.00683E-12 |
| F2RL1 | 10.49806 | 10.59932 | 10.71069 | 11.1392 | 11.24369 | 11.07512 | -2.055635 | 1.45E-15 |
| POLR3G | 10.97836 | 11.03628 | 11.10674 | 11.94257 | 11.9041 | 11.93197 | -2.046398 | 2.25E-56 |
| RGS4 | 10.79634 | 10.81867 | 10.77335 | 11.45055 | 11.46429 | 11.50798 | -2.037277 | 5.76E-42 |
